# Effect of experimental and clinical pain on the spatial distribution of muscle activity: a systematic review and meta-analysis

**DOI:** 10.3389/fnhum.2025.1603807

**Published:** 2025-07-10

**Authors:** Guillermo Mendez-Rebolledo, Ignacio Orozco-Chavez, Joaquín Salazar-Méndez, Juan Morales-Verdugo, Eduardo Martinez-Valdes

**Affiliations:** ^1^Laboratorio de Investigación Somatosensorial y Motora, Escuela de Kinesiología, Facultad de Salud, Universidad Santo Tomás, Talca, Chile; ^2^Departamento de Ciencias del Movimiento Humano, Facultad de Ciencias de la Salud, Universidad de Talca, Talca, Chile; ^3^Departamento de Ciencias Preclínicas, Facultad de Medicina, Universidad Católica del Maule, Talca, Chile; ^4^School of Sport, Exercise and Rehabilitation Sciences, College of Life and Environmental Sciences, University of Birmingham, Birmingham, United Kingdom

**Keywords:** clinical pain, experimental pain, regional muscle activity, electromyography, high density surface electromyography, neuromuscular adaptation

## Abstract

**Introduction:**

Musculoskeletal dysfunctions can significantly impair quality of life due to persistent pain and neuromuscular adaptations. While regional activation patterns in healthy muscles are well-documented, the effects of clinical and experimental pain on these patterns remain inconsistent. Accordingly, this study systematically evaluates the scientific evidence on alterations in the spatial distribution of muscle activity, quantified by shifts in the center of activity of high-density surface electromyography (HD-sEMG) signals, under experimental and clinical pain conditions.

**Methods:**

A comprehensive database search was conducted from inception to June 6, 2025. The review included studies that evaluated the spatial distribution of muscle activity with HD-sEMG, analyzing two-dimensional shifts in the center of activity among individuals with clinical or experimentally induced pain. Methodological quality was assessed using the adapted Newcastle-Ottawa Scale, and evidence certainty was evaluated with the GRADE approach. A random-effects model was employed in the meta-analysis to account for variability across studies.

**Results:**

Twenty studies involving 562 participants (231 control, 266 clinical pain, and 65 experimental pain) were included. The meta-analysis revealed a statistically significant shift in the center of activity in individuals with clinical pain compared with asymptomatic controls (SMD = 0.49; 95% CI = 0.15 to 1.84; *p* = 0.004), particularly those with chronic low back pain, with a low effect size (SMD = 0.43; 95% CI = 0.03 to 0.83; *p* = 0.04), indicating altered spatial distribution of muscle activity. A meta-analysis for experimental pain was not feasible due to limited data.

**Conclusions:**

These findings underscore that clinical pain is associated with altered spatial distribution of muscle activity and emphasize the need for standardized methodologies and further research across diverse populations to enhance pain management and rehabilitation strategies.

**Systematic review registration:**

This study was prospectively registered in the International Prospective Register of Systematic Reviews (PROSPERO) (identifier CRD42024534320), https://www.crd.york.ac.uk/PROSPERO/view/CRD42024534320.

## 1 Introduction

For chronic musculoskeletal dysfunctions, such as patellofemoral pain syndrome, chronic ankle instability, and chronic low back pain (CLBP), significantly impact global health, leading to reduced quality of life and increased healthcare expenditures (Chia et al., 2022; Perrot et al., 2022; Hong and Calder, 2023). These conditions are characterized by persistent or chronic pain, leading to non-stereotypical neuromuscular adaptations (Graven-Nielsen, 2022; Devecchi et al., 2023). These adaptations manifest as changes in range of motion, movement variability, amplitude and distribution of muscle activity, timing of muscle activity, and corticospinal excitability (Behnke et al., 2021; Devecchi et al., 2023; Rogoschin et al., 2024). According to current theories, pain does not uniformly affect groups of motoneurons but instead causes non-uniform effects on motoneuron pools, leading to a redistribution of activity within (i.e., altered regional activation) or between muscles (Hodges et al., 2021; Dernoncourt et al., 2025; Hug et al., 2025). It has been suggested that this adaptation aims to protect the painful area from further injury (Hodges and Tucker, 2011; Hodges and Smeets, 2015; Hodges et al., 2021).

High-density surface electromyography (HD-sEMG) has emerged as a valuable technique to overcome the limitations of traditional single-channel sEMG, which lacks the spatial resolution needed to detect regional muscle activation patterns (Besomi et al., 2020; Gallina et al., 2022; Mendez-Rebolledo et al., 2023b). HD-sEMG, which uses a grid of electrodes, enhances spatial resolution and allows for more precise mapping of muscle activity (Drost et al., 2006). A widely used analysis method is the center of activity, also referred to as the centroid, barycenter, or center of mass, which summarizes the distribution of sEMG amplitude across the electrode grid as x- and y-coordinates (Gallina et al., 2022). This center shifts during motor tasks and contractions, reflecting relative changes in the spatial location of activation. Displacements of the center of activity have been reported in muscles such as the pectoralis major (Cabral et al., 2022), erector spinae (Arvanitidis et al., 2021), vastus medialis (Gallina et al., 2019), and fibularis longus (Mendez-Rebolledo et al., 2021a), suggesting spatial reorganization of muscle activity in different tasks. The center of activity, while susceptible to artifacts such as electrode shift, cross-talk, and cardiovascular noise (Farina et al., 2004), is a commonly used measure in HD-sEMG research. Although its validity has not been systematically established, it is recommended in recent methodological guidelines for spatial EMG analysis (Gallina et al., 2022). While motor unit decomposition provides greater mechanistic insight, its application is more feasible during isometric or less demanding motor tasks, as it is highly sensitive to movement artifact and signal noise (Martinez-Valdes et al., 2023). In contrast, the center of activity remains commonly used in dynamic, functionally demanding tasks involving complex muscles like the erector spinae (e.g., lumbar endurance and lifting activities) (Arvanitidis et al., 2021; Sanderson et al., 2024). Given the heterogeneity of protocols in pain-related research, this method offers a practical and comparable approach for assessing spatial muscle activation across studies.

Despite the high prevalence of chronic musculoskeletal dysfunctions (Hiller et al., 2012), it remains unclear whether clinical pain consistently alters the spatial distribution of muscle activity, as previous studies have yielded conflicting results. For instance, Gallina et al. (2019) used HD-sEMG to examine vasti muscle activation in females with patellofemoral pain and found that healthy individuals displayed more complex spatial patterns, requiring a greater number of principal components to explain signal variance. In contrast, individuals with pain exhibited reduced spatial complexity and intermuscular coordination, suggesting a less adaptable motor strategy (Gallina et al., 2019). Even within a single contraction, shifts in the center of activity may reflect altered motor unit recruitment patterns associated with chronic pain. Although such neuromuscular adaptations may already be established, comparing spatial activation between patients and healthy controls remains essential. It enables the identification of reorganization patterns, quantification of their magnitude, and detection of potentially maladaptive spatial patterns. These insights are critical for informing targeted rehabilitation strategies. Similarly, experimental pain models, such as infrapatellar hypertonic saline injections, have shown reduced activation in the distal regions of the vastus medialis and lateralis (Gallina et al., 2018b). Conversely, other studies have reported a uniform activation pattern of the vastus medialis under induced pain (Hug et al., 2014a,b), highlighting the methodological variability across investigations. Such inconsistencies may result from methodological differences, variations in motor tasks, or the pain models used, and highlight the need to guide future research toward identifying spatial activation patterns that may be maladaptive and contribute to persistent dysfunction (Gallina et al., 2018b).

Current evidence indicates that pain may alter motor unit recruitment and regional activation patterns, changing the spatial distribution of force within the muscle. These changes can influence the orientation of the resulting joint force vector (Tucker and Hodges, 2010; Gallina et al., 2018b). However, it remains unclear whether these changes are primarily driven by experimental pain, clinical pain, or a combination of both (Hodges and Tucker, 2011; Hug et al., 2014b; Gallina et al., 2018b; Hodges et al., 2021). In addition, pain-related alterations in muscle activity may also be task-specific. These alterations may serve as adaptive strategies to mitigate pain, protect the affected area, and delay fatigue during repetitive tasks. Therefore, this study systematically evaluates the scientific evidence on alterations in the spatial distribution of muscle activity, quantified by shifts in the center of activity of HD-sEMG signals, under experimental and clinical pain conditions. Synthesizing these findings will provide an overview of how pain is reflected in spatial shifts of muscle activity, highlight methodological strengths and limitations, and outline directions for mechanistic research that could ultimately inform future treatment and rehabilitation strategies.

## 2 Methods

This systematic review and meta-analysis were conducted following the PRISMA and MOOSE reporting guidelines (Stroup, 2000; Page et al., 2021). This study was prospectively registered in the International Prospective Register of Systematic Reviews (PROSPERO) (CRD42024534320).

### 2.1 Data sources and searches

The search strategy was applied in PubMed/MEDLINE, Web of Science, Scopus, CINAHL, and SPORTDiscus using a combination of Medical Subject Headings terms, keywords, and Boolean operators. Specific search terms and combinations can be found in Table 1. In addition, the reference lists of eligible articles were manually searched in Google Scholar, and experts in the field were consulted to identify studies that were not found with the search strategy.

**Table 1:** Search strategy.

**Data base**	**Search strategy**	**Result**
MEDLINE	(((((Regional activation) OR (Regional activity)) OR (Regional myoelectric activity)) AND ((((High-density electromyography) OR (High-density surface electromyography)) OR (Multichannel surface electromyography)) OR (Electromyography))) AND ((((((injuries) OR (injury)) OR (pain)) OR (musculoskeletal injuries)) OR (musculoskeletal injury)) OR (musculoskeletal diseases))) NOT ((((((cerebral stroke) OR (spinal cord injury)) OR (nerve injury)) OR (root nerve)) OR (neuropathy)) OR (neurological disease))	881
Scopus	ALL ((((((“Regional activation”) OR (“Regional activity”)) OR (“Regional myoelectric activity”)) AND ((((“High-density electromyography”) OR (“High-density surface electromyography”)) OR (“Multichannel surface electromyography”)) OR (electromyography))) AND ((((((injuries) OR (injury)) OR (pain)) OR (“musculoskeletal injuries”)) OR (“musculoskeletal injury”)) OR (“musculoskeletal diseases”))) AND NOT ((((((“cerebral stroke”) OR (“spinal cord injury”)) OR (“nerve injury”)) OR (“root nerve”)) OR (neuropathy)) OR (“neurological disease”)))	152
Web of Science	#1 ((TS=(regional activation)) OR TS=(regional activity)) OR TS=(regional myoelectric activity) #2 (((TS=(High-density electromyography)) OR TS=(High-density surface electromyography)) OR TS=(multichannel surface electromyography)) OR TS=(electromyography) #3 (((((TS=(injuries)) OR TS=(injury)) OR TS=(pain)) OR TS=(musculoskeletal injuries)) OR TS=(musculoskeletal injury)) OR TS=(musculoskeletal diseases) #4 (((((TS=(cerebral stroke)) OR TS=(spinal cord injury)) OR TS=(nerve injury)) OR TS=(root nerve)) OR TS=(neuropathy)) OR TS=(neurological disease) #5 #1 AND #2 AND #3 NOT #4	147
SPORT Discus	(Regional activation) OR (Regional activity) OR (Regional myoelectric activity) AND (High-density electromyography) OR (High-density surface electromyography) OR (Multichannel surface electromyography) OR (Electromyography) AND (injuries) OR (injury) OR (pain) OR (musculoskeletal injuries) OR (musculoskeletal injury) OR (musculoskeletal diseases) NOT (cerebral stroke) OR (spinal cord injury) OR (nerve injury) OR (root nerve) OR (neuropathy) OR (neurological disease)	22
CINAHL	(Regional activation) OR (Regional activity) OR (Regional myoelectric activity) AND (High-density electromyography) OR (High-density surface electromyography) OR (Multichannel surface electromyography) OR (Electromyography) AND (injuries) OR (injury) OR (pain) OR (musculoskeletal injuries) OR (musculoskeletal injury) OR (musculoskeletal diseases) NOT (cerebral stroke) OR (spinal cord injury) OR (nerve injury) OR (root nerve) OR (neuropathy) OR (neurological disease)	12
Total		1214

### 2.2 Eligibility criteria

The PECO framework was used as inclusion criteria (Populations, Exposures, Comparators and Outcomes) (Morgan et al., 2018; Dekkers et al., 2019): (i) populations: any human subject; (ii) exposures: any musculoskeletal chronic condition associated to clinical pain or experimentally induced pain; (iii) comparators: a non-exposed reference population, which includes healthy individuals with no history of musculoskeletal disease or dysfunction in the last 6 months, or healthy individuals serving as their own control when comparing results before (baseline) and after exposure to experimental pain (e.g., hypertonic saline injection); (iv) outcomes: spatial distribution of muscle activity, defined as the relative localization sEMG amplitude across a muscle (Gallina et al., 2022). This distribution can be assessed either by comparing the amplitude of the sEMG signal—such as root mean square (RMS) or average rectified value (ARV)—in specific regions, or by analyzing changes across multiple sEMG electrodes (i.e., HD-sEMG arrays). The latter approach involves computing displacements in the center of activity (also referred to as barycenter, centroid, center of mass, or locus) to summarize spatial shifts in muscle activity (Gallina et al., 2022). Cross-sectional studies of peer-reviewed articles written in English or Spanish, published from inception to June 6, 2025, were included. Exclusion criteria for this study were: (i) research on neurological disease; (ii) all editorials, letters, reviews, and meta-analyses.

### 2.3 Study selection

Two independent reviewers (IO-C and JM-V) used Rayyan web software (http://rayyan.qcri.org) to analyze the results (Ouzzani et al., 2016). After removing duplicates, studies were selected by title and abstract. Those potentially eligible studies were read in full text, and the inclusion and exclusion criteria were applied. In case of disagreement during any of the phases, a third author was consulted to resolve (GM-R).

### 2.4 Data collection

A standardized table was used for data collection. Two independent reviewers (IO-C and JM-V) extracted data from the studies. In case of disagreement, a third reviewer (GM-R) resolved the disagreement. Data collected for each study included: author, muscle group and electrode location, signal derivation and electrode specifications, sample size, sex, sEMG outcomes, task, pain intensity, and spatial distribution results. For studies with missing data, we attempted to contact the authors 3 times, by email.

### 2.5 Risk of bias assessment

The Newcastle-Ottawa Scale (NOS), adapted for this study, was utilized to assess the methodological quality of cross-sectional studies (Modesti et al., 2016). Previous systematic reviews on observational studies involving sEMG have employed this scale (Mendez-Rebolledo et al., 2021b). The adapted NOS comprises seven items, focusing on sample selection, comparability, and outcome. Each subitem is rated from 0 to 2 stars, with a maximum total score of 9. The comparability item examines the control of potential confounding factors. A single star is awarded when the study considered confounders related to the presence of clinical or experimental pain and conducted subgroup analyses accordingly (e.g., by pain type, pain location, or interaction with contraction type or movement phase). Methodological quality was classified using established thresholds from prior systematic reviews (Modesti et al., 2016; Mendez-Rebolledo et al., 2021b, 2022): studies scoring 0–4 stars were rated as low quality, 5–7 as moderate quality, and 8–9 as high quality. Discrepancies in scores will be resolved through consensus, and the agreed-upon rating will be assigned to each study. The reviewers must achieve substantial agreement (kappa coefficient ≥ 0.80) in the final classification of the studies. Additionally, an adaptation of the Consensus for Experimental Design in Electromyography (CEDE) checklist was implemented to assess the methodological quality and reporting transparency of the sEMG procedures employed in the included studies (Besomi et al., 2024). This checklist originally contained 40 items, divided into two sections. For this study, only the ‘Procedure for sEMG Recording' section was considered. Items that were not applicable due to study design or the type of electromyography (i.e., needle or wireless) were excluded, resulting in a modified 20-point checklist.

### 2.6 Certainty of evidence

The Grading of Recommendations Assessment, Development, and Evaluation (GRADE) approach was used to grade the certainty of the evidence for each outcome (Guyatt et al., 2008). Two reviewers (J S-M and G M-R) used GRADEpro (https://gradepro.org) to produce a summary table of results. The certainty of the evidence was determined in two stages. In the first, it was considered to reduce the certainty according to the following criteria: (i) limitation of the included studies: decrease one level if 25% or more of the included articles had a high risk of bias evaluated with NOS; (ii) inconsistency: decrease one level if there was high heterogeneity (I^2^ ≥ 75%); (iii) indirectness: down one level if there were differences between participants, interventions, outcome measures or indirect comparisons; (iv) imprecision: a markdown level was considered if there was a wide confidence interval, crosses the line of no effect, and small sample size (*n* < 300); (v) risk of publication bias: decrease one level if there was asymmetry in the funnel plot.

### 2.7 Data analysis

ReviewManager version 5.3 (The Nordic Cochrane Center, The Cochrane Collaboration) was used for statistical analysis. The standardized mean difference (SMD) (Anzures-Cabrera et al., 2011) and 95% confidence intervals (CIs) were calculated to estimate the differences in regional activity (e.g., center of activity) between patients with clinical pain (musculoskeletal injuries), experimental pain, and a combination of both, compared to healthy controls. When the standard deviation (SD) was not reported by the studies, standard formulas were used to derive it based on the standard error (SE), the 95% CI, or the *p*-value of a *t*-test (Deeks et al., 2021). Studies were pooled using a random-effects model with the DerSimonian and Laird method, as heterogeneity in true effect sizes was assumed between included studies (Borenstein et al., 2010). An SMD of 0.0 to 0.2 represented a trivial effect, 0.2 to 0.6 a small effect, 0.6 to 1.2 a moderate effect, 1.2 to 2.0 a large effect, 2.0–4.0 a very large effect, and 4.0 an extremely large effect (Hopkins et al., 2009). Heterogeneity was assessed using the I^2^ statistic, considering values of < 25% as low, 25%−75% as moderate, and >75% as high heterogeneity (Higgins, 2003). In addition, if there was a high level of heterogeneity (i.e. I^2^ > 75%), a sensitivity analysis was applied to remove one study at a time to determine the impact on the heterogeneity of the results (Higgins, 2003).

## 3 Results

### 3.1 Study selection

**Figure 1:**
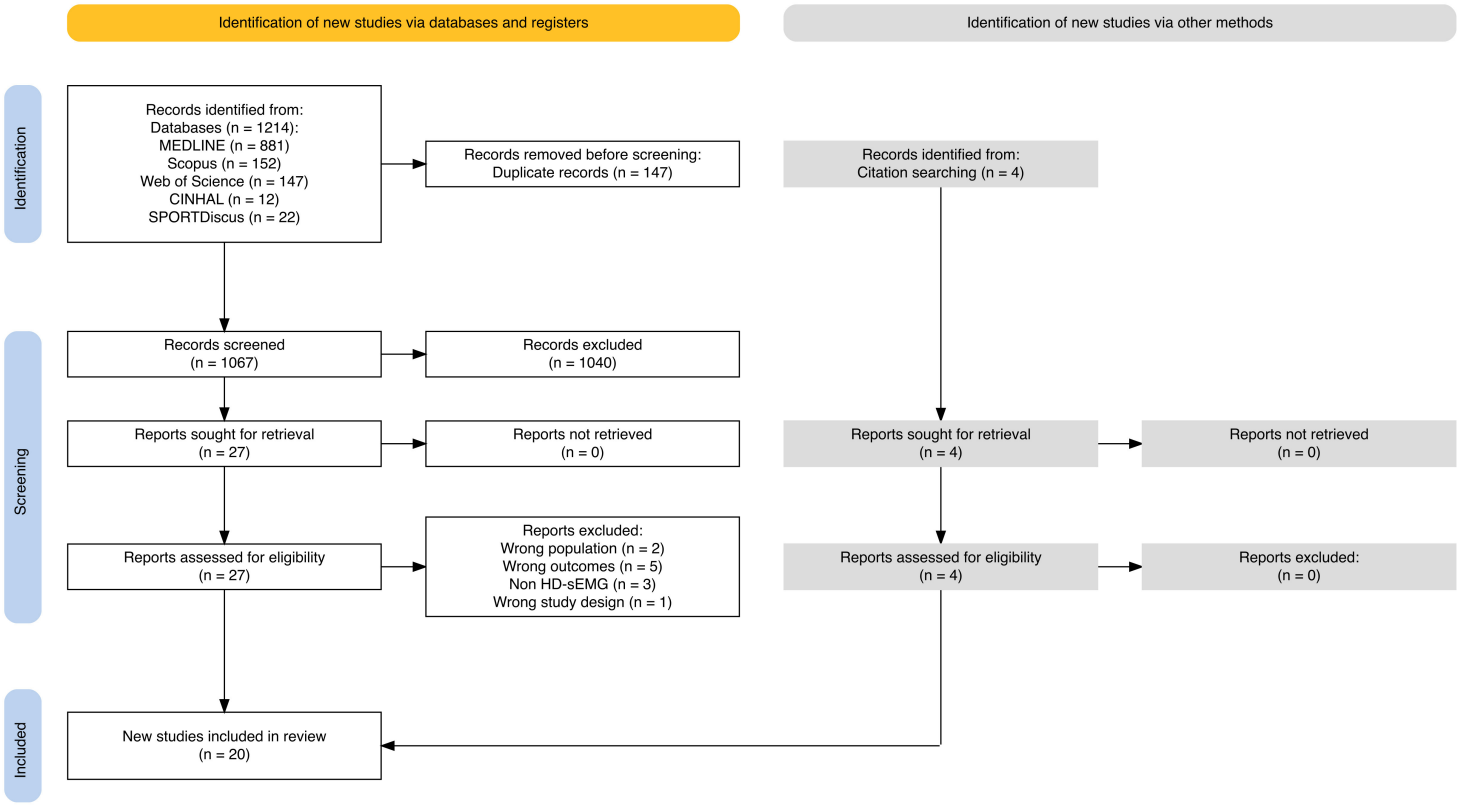
PRISMA flow diagram. PRISMA, preferred reporting items for systematic reviews and meta-analyses. HD-sEMG, high-density surface electromyography.

The results of the search are reported in Figure 1. In total, 1,214 articles were identified from databases. After the removal of duplicates (*n* = 147), 1,067 articles were screened by title and abstract, excluding 1,040 articles. The remaining 27 articles were included in the review process. Eleven articles were excluded due to outcomes not aligned with the review's focus on the spatial distribution of muscle activity (Supplementary Table S1). The reasons for exclusion were as follows: the absence of center of activity or comparable spatial analyses (*n* = 5) (Finneran et al., 2003; Yong et al., 2004; Gaudreault et al., 2005; Sung et al., 2005; Gallina et al., 2018a), use of non-HD-sEMG systems or the absence of a multichannel electrode configuration capable of spatially sampling muscle activity (*n* = 3) (Pirouzi et al., 2006; Schabrun et al., 2017; Claus et al., 2018), non-eligible populations (*n* = 2) (Kubo et al., 2019; Abboud et al., 2021), and studies applying therapeutic interventions that may have influenced the spatial distribution of muscle activity (*n* = 1) (Mendez-Rebolledo et al., 2025). Additionally, four articles were identified from reference citations, including a total of twenty articles in this review (Figure 1), of which fifteen were included in the meta-analysis based on available quantitative data on center of activity displacement.

### 3.2 Characteristics of the studies

The characteristics of the 20 studies included in this review are summarized in Tables 2, 3. The sample was made up of 562 participants [231 control (healthy); 266 clinical pain; 65 experimental pain], including 245 females and 317 males. The reported age ranged from 17.8 to 46.6 years, with a pooled mean age of 30.5 ± 6.7 years. Four musculoskeletal dysfunctions associated with clinical pain were identified in the included studies: CLBP (*n* = 12) (Abboud et al., 2014; Falla et al., 2014; Martinez-Valdes et al., 2019; Sanderson et al., 2019a,b, 2024; Hao et al., 2020; Arvanitidis et al., 2021, 2022, 2023; Serafino et al., 2021; Sampieri et al., 2025), patellofemoral pain syndrome (*n* = 1) (Gallina et al., 2019), and chronic ankle instability (*n* = 1) (Mendez-Rebolledo et al., 2023a), and lumbar myofascial trigger point (n = 1) (Li et al., 2024). Two experimental pain conditions were identified: upper trapezius pain by hypertonic saline injection (*n* = 4) (Madeleine et al., 2006; Dideriksen et al., 2016; Falla et al., 2017; Ducas et al., 2024) and erector spinae pain by nociceptive electrical stimulation (*n* = 1) (Ducas et al., 2024). Neuromuscular activity was measured across different tasks and differentiated by the type of muscle contraction involved. This allowed the same study to provide information on both the concentric and eccentric phases when dynamic tasks were performed. Neuromuscular activity was measured during isometric (*n* = 11) (Madeleine et al., 2006; Abboud et al., 2014; Dideriksen et al., 2016; Gallina et al., 2018b; Sanderson et al., 2019b; Hao et al., 2020; Arvanitidis et al., 2022; Mendez-Rebolledo et al., 2023a; Ducas et al., 2024; Li et al., 2024; Sampieri et al., 2025), concentric (*n* = 9) (Falla et al., 2014, 2017; Gallina et al., 2019; Martinez-Valdes et al., 2019; Sanderson et al., 2019a, 2024; Arvanitidis et al., 2021, 2023; Serafino et al., 2021), and eccentric (*n* = 8) (Falla et al., 2014, 2017; Gallina et al., 2019; Martinez-Valdes et al., 2019; Sanderson et al., 2019a, 2024; Serafino et al., 2021; Arvanitidis et al., 2023) tasks.

**Table 2:** Characteristics of the included articles: clinical pain.

**References**	**Muscle group and electrode location**	**Signal derivation and electrode specifications**	**Group**	**N (F/M)**	**Age (Mean ±SD)**	**sEMG outcomes**	**Task**	**Average pain (Mean ±SD)**	**Spatial distribution results**
Abboud et al. (2014)	Lumbar Erector Spinae 2 cm lateral to L3 spinous process	Signal Derivation: Bipolar Grid Dimension:13 rows × 5 columns Inter-electrode distance: 12.5 mm	CTR	23 (10/13)	37.8 ± 10.3	RMS dispersion (mm)	Isometric trunk extension endurance task at 30% MVC	VAS Basal = 0 VAS Final = 0	Lower RMS dispersion in CLBP
			CLBP	46 (19/27)	43.7 ± 13.6			VAS Basal = 16.9 ± 17.3 VAS Final = 29.5 ± 20.9	
Arvanitidis et al. (2021)	Lumbar Erector Spinae 2 cm lateral to L5 spinous process	Signal Derivation: Monopolar Grid Dimension: 13 rows × 5 columns Inter-electrode distance: 8 mm	CTR	11 (6/5)	26.4 ± 5.5	Y-axis barycenter (mm) and RMS (%)	Extension phase (CON) of an Isokinetic trunk flexion-extension task at 60°/s	VAS Basal = 0 VAS Final = 0	Barycenter shifted cranially in individuals with CLBP after completing the task
			CLBP	12 (6/6)	25.9 ± 9.7			VAS Basal = 1.9 ± 2.1 VAS Final = 3.4 ± 2.5	
Arvanitidis et al. (2022)	Lumbar Erector Spinae 2 cm lateral to L5 spinous process	Signal Derivation: Monopolar Grid Dimension: 13 rows × 5 columns Inter-electrode distance: 8 mm	CTR	15 (7/8)	27.4 ± 4.9	RMS (%) and Coherence	Isometric trunk extension endurance task at 20% and 50% MVC	NPRS = 0	EMG-torque coherence shifted cranially at 50%MVC in the CLBP group
			CLBP	15 (8/7)	27.1 ± 9.3			NPRS = 2.5 ± 2.2	
Falla et al. (2014)	Lumbar erector spinae 2 cm lateral to L5 spinous process	Signal derivation: bipolar Grid dimension: 13 rows × 5 columns Inter-electrode distance: 8 mm	CTR	17 (8/9)	29.4 ± 7.4	RMS (%) and Y-axis centroid (mm)	Lumbar extension (CON) and flexion (ECC) during box lifting and lowering task	NPRS Basal = 0 NPRS Final = 0	Barycenter shifted cranially in individuals with CLBP after completing the task
			CLBP	19 (11/8)	32.2 ± 9.5			NPRS Basal = 1.8 ± 0.4 NPRS Final = 2.6 ± 0.5	
Hao et al. (2020)	Lumbar erector spinae 2 cm lateral to l2-l5 spinous process	Signal derivation: monopolar Grid dimension: 8 rows × 2 columns Inter-electrode distance: horizontal 7.5 mm vertical 10.05 mm	CTR	20 (0/20)	29.0 ± 4.6	RMS dispersion (mm), Y-axis centroid, and entropy	Isometric trunk extension task (1-min Sorensen test)	VAS = 0	Barycenter shifted cranially in individuals with CLBP after completing the task
			CLBP	20 (0/20)	28.6 ± 3.8			VAS = 4.2 ± 1.5	
Sanderson et al. (2019a)	Thoracolumbar Erector Spinae 2 cm lateral to T8-L5 spinous process	Signal derivation: monopolar Grid dimension: 13 rows × 5 columns Inter-electrode distance: 8 mm	CTR	14 (6/8)	27.3 ± 11.3	RMS (mV), Y-axis centroid, and entropy	Lumbar extension (CON) and flexion (ECC) during box lifting and lowering task	NPRS Average = 0	Barycenter shifted cranially in individuals with CLBP after completing the task
			CLBP	11 (6/5)	32.4 ± 16.2			NPRS Average = 3.3 ± 2.0	
Sanderson et al. (2019b)	Lumbar Erector Spinae 2 cm lateral to L3-L5 spinous process	Signal Derivation: Monopolar Grid Dimension: 13 rows × 5 columns Inter-electrode distance: 8 mm	CTR	13 (6/7)	26.4 ± 5.0	RMS (mV) and Y-axis centroid	Isometric trunk extension endurance task (prone position)	NPRS Average = 0	Barycenter shifted cranially in individuals with CLBP after completing the task
			CLBP	13 (7/6)	39.0 ± 9.7			NPRS Average = 2.9 ± 1.9	
Martinez-Valdes et al. (2019)	Lumbar Erector Spinae (Iliocostalis) 2 cm lateral to L3-L5 spinous process	Signal Derivation: Monopolar Grid Dimension: 13 rows × 5 columns Inter-electrode distance: 8 mm	CTR	10 (2/8)	27.0 ±14.3	RMS (mV), Y-axis centroid, and entropy	Incremental extension (CON) and flexion (ECC) rowing task	NR	Barycenter shifted caudally in individuals with CLBP after completing the task
			CLBP	8 (3/5)	32.1 ± 17.6			NR	
Sampieri et al. (2025)	Lumbar Erector Spinae (Iliocostalis) 2 cm lateral to L3-L5 spinous process	Signal Derivation: Monopolar Grid Dimension: 13 rows × 5 columns Inter-electrode distance: 8 mm	CTR	11 (1/10)	37.3 ± 13.1	RMS (%), Y-axis barycenter, and entropy	Isometric trunk position during an incremental cycling test (70% to 100%)	CGPQ = 0	Lower entropy values in LBP as the intensity increased.
			CLBP	10 (0/10)	42.2 ±11.9			CGPQ (Pain intensity) = 33.3 ± 4.4	
Sanderson et al. (2024)	Lumbar Erector Spinae 2 Grids bilaterally Lower grids 2 cm lateral to L5 spinous process, upper grids 5 mm cranial to lower	Signal Derivation: Monopolar Grids Dimension: 13 rows × 5 columns Inter-electrode distance: 8 mm	CTR	15 (9/6)	26.87 ± 11.13	RMS (%), Y-axis barycenter, and entropy	Lumbar extension (CON) and flexion (ECC) during repeated box lifting in a three dimensional, multiplanar manner.	NPRS Current = 0 NPRS Average = 0	Barycenter shifted cranially in individuals with CLBP across all cycles of movement.
			LBP	14 (7/7)	32.14 ± 14.64			NPRS Current = 2.68 ± 2.03 NPRS Average= 5.93 ± 1.69	
Li et al. (2024)	Lumbar Erector Spinae 2 cm lateral to L2, from T12 to L4	Signal Derivation: NR Grid Dimension: 8 rows × 5 columns Inter-electrode distance: 8.5 mm	CTR	3 (0/3)	43.6 ± 7.2	RMS, centroid of the low-energy region	Isometric trunk extension	NR	The centroid of low energy was close to LMTrP. Muscle activity changed more significantly in healthy individuals.
			LMTrP	3 (0/3)	46.6 ± 5.2			NR	
Serafino et al. (2021)	Thoracolumbar Erector Spinae 2 to 3.5 cm lateral to T8-L5 spinous process	Signal Derivation: Monopolar Grid Dimension: 16 rows × 2 columns Inter-electrode distance: Horizontal 10 mm Vertical 15 mm	CTR	21 (11/10)	39.3 ± 13.5	Amplitude (μV) and location (EMG channel number)	Stand-up (CON) and sit-down (ECC) phases of Sit-to-stand test	NPRS = 0	Barycenter did not show a significant shift in individuals with CLBP after completing the task
			CLBP	21 (11/10)	43.5 ± 12.5			NPRS = 4.4 ± 1.5	
Arvanitidis et al. (2023)	Thoracolumbar Erector Spinae 2 cm lateral to T10-L5 spinous process	Signal Derivation: Monopolar Grids Dimension: 13 rows × 5 columns Inter-electrode distance: 8 mm	CTR	20 (10/10)	28.6 ± 3.9	Y-axis barycenter (mm)	Extension (CON) and flexion (ECC) trunk task at 25% and 50% MVC	NPRS = 0.6 ± 0.8	Barycenter did not show significant shift in individuals with CLBP after completing the task
			CLBP	20 (10/10)	31.1 ± 6.9			NPRS = 4.6 ± 1.7	
Gallina et al. (2019)	Vastus Medialis Vastus lateralis The grid's center was positioned at 50% between the medial and lateral borders of each muscle	Signal Derivation: Monopolar Grid Dimension: 16 rows × 1 columns Inter-electrode distance: 10 mm	CTR	20 (20/0)	26.0 ± 4.0	*Number of PC* *Spatial weights* PC1 General activation PC2 Vastus specific activation PC3 Vasti coactivation PC4 Proximal-distal Vasti coactivation *Temporal coefficients* PC1 and PC2	Knee flexion (ECC)-extension (CON) performed within from approximately 100° to 5° against resistance	NPRS = 0	A lower number of PCs and no regional activation was observed in the Vastus Medialis in the Spatial weight analysis in individuals with PFPS
			PFPS	36 (36/0)	27.0 ± 4.0			NPRS = 4.1 ± 1.3	
Mendez-Rebolledo et al. (2023a)	Fibularis Longus The grid's center was positioned at 32% between the top of the head and the lateral malleolus	Signal Derivation: Monopolar Grid Dimension: 13 rows × 5 columns Inter-electrode distance: 2.5 mm	CTR	18 (0/18)	18.0 ± 1.5	RMS (%) of anterior and posterior compartments; X- and Y-axis center of mass	Isometric eversion at different force levels	NPRS = 0.18 ± 0.53	The center of mass shifted anteriorly in individuals with CAI after completing the task
			CAI	18 (0/18)	17.8 ± 1.5			NPRS = 0.24 ± 0.56	

sEMG, surface electromyography; F/M, female/male; CTR, control; CLBP, chronic low back pain; RMS, root mean square; MVC, maximum voluntary isometric contraction; CON, concentric task; ECC, eccentric task; VAS, visual analog scale; NPRS, numerical pain rating scale; NR, Not reported; CGPQ, chronic pain grade questionnaire; LMTrP, Lumbar Miofrascial Trigger Point; PC, principal component; PFPS, patellofemoral pain syndrome; CAI, chronic ankle instability.

**Table 3:** Characteristics of included articles: experimental pain.

**References**	**Muscle group and Electrode Location**	**Signal derivation and Electrode specifications**	**Group**	**N (F/M)**	**Age (Mean ±SD)**	**sEMG Outcomes**	**Task**	**Average pain (Mean ±SD)**	**Spatial distribution results**
Ducas et al. (2024)	Lumbar Erector Spinae 1 cm lateral to L3 spinous process	Signal Derivation: Bipolar Grid Dimension: 8 rows × 8 columns Inter-electrode distance: 10 mm	CTR	19 (9/10)	25.3 ± 4.7	RMS (%) and Y-axis centroid (mm)	Isometric trunk extension in different positions: neutral, 45° flexion, and 90° flexion	NPRS Neutral = 0 NPRS 45° flexion *=* 0 NPRS 90° flexion = 0	Centroid did not show a significant shift in individuals exposed to experimental pain after completing the task
			PAIN: NES	19 (9/10)	25.3 ± 4.7			NPRS Neutral = 3.1 ± 0.5 NPRS 45° flexion = 3.0 ± 0.4 NPRS 90° flexion = 2.9 ± 0.6	
Dideriksen et al. (2016)	Upper Trapezius 4^th^ row of the grid along the C7–acromion line	Signal Derivation: Bipolar Grid Dimension: 13 rows × 5 columns Inter-electrode distance: 8 mm	CTR	12 (6/6)	26.5 ± 5.1	Y-axis barycenter (mm)	Isometric shoulder abduction (90°) for 60 seconds	NPRS = 0	Barycenter shifted caudally in individuals exposed to experimental pain after completing the task
			PAIN: HSI	12 (6/6)	26.5 ± 5.1			NPRS Cranial = 4.2 ± 1.8 NPRS Caudal = 4.8 ± 1.6	
Falla et al. (2017)	Upper Trapezius 4^th^ row of the grid along the C7–acromion line	Signal Derivation: Bipolar Grid Dimension: 13 rows × 5 columns Inter-electrode distance: 8 mm	CTR	10 (0/10)	26.2 ± 3.1	RMS (%), Y-axis barycenter (mm), and entropy	1 Kg box lifting (CON) and lowering (ECC) task	NPRS = 0 NPRS ISI = 0.9 ± 0.8	Barycenter shifted caudally in individuals exposed to experimental pain after completing the task
			PAIN: HSI	10 (0/10)	26.2 ± 3.1			NPRS HSI = 5.5 ± 1.8	
Madeleine et al. (2006)	Upper Trapezius 4^th^ row of the grid along the C7–acromion line	Signal Derivation: Bipolar Grid Dimension: 13 rows × 5 columns Inter-electrode distance: 5 mm	CTR	10 (0/10)	23.9 ± 1.9	Y-axis barycenter	Isometric shoulder abduction (90°) for 90 seconds	NPRS = 0	Barycenter shifted caudally in individuals exposed to experimental pain after completing the task
			PAIN: HSI	10 (0/10)	23.9 ± 1.9			NPRS HSI = 5.0 ± 0.5	
Gallina et al. (2018b)	Vastus Medialis and Vastus Lateralis Aligned to innervation zone	Signal Derivation: Monopolar Grid Dimension: 13 rows × 5 columns Inter-electrode distance: 8 mm	CTR	14 (7/7)		ARV Y-axis barycenter	Isometric knee extension at 10% MVC	NPRS = 0	Less activation of the VMD in individuals exposed to VM or VL experimental pain
			PAIN: HSI	14 (7/7)				NPRS FP = 2.9 ± 1.1 NPRS VMD = 3.4 ± 1.2 NPRS VMP = 3.3 ± 1.1 NPRS VL = 3.1 ± 1.3	

sEMG, surface electromyography; F/M, female/male; CTR, control; NES, nociceptive electrical stimulation; HSI, hypertonic saline injection; RMS, root mean square; NPRS, numerical pain rating scale; CON, concentric task; ECC, eccentric task; ISI, isotonic saline injection; AVR, average rectified value; FP, infrapatellar fat pad; VMD, distal vastus medialis; VMP, proximal vastus medialis; VL, vastus lateralis; VM, vastus medialis.

Regarding the spatial distribution of muscle activity, it was primarily characterized by the displacement of the center of activity (barycenter, center of mass, centroid, or locus) along the cephalocaudal axis (Y-axis) (*n* = 16) (Madeleine et al., 2006; Falla et al., 2014, 2017; Dideriksen et al., 2016; Gallina et al., 2019; Martinez-Valdes et al., 2019; Sanderson et al., 2019a,b, 2024; Hao et al., 2020; Arvanitidis et al., 2021, 2023; Mendez-Rebolledo et al., 2023a; Ducas et al., 2024; Li et al., 2024; Sampieri et al., 2025). In addition to center-of-activity analyses, several studies included other spatial or signal-based sEMG outcomes. Two studies assessed RMS dispersion (Abboud et al., 2014; Hao et al., 2020), one study performed coherence analysis (Arvanitidis et al., 2022), and one study used principal component analysis (Gallina et al., 2019). Additionally, several studies reported amplitude-based outcomes, including RMS (*n* = 12) (Falla et al., 2014, 2017; Martinez-Valdes et al., 2019; Sanderson et al., 2019a,b, 2024; Arvanitidis et al., 2021, 2022; Mendez-Rebolledo et al., 2023a; Ducas et al., 2024; Li et al., 2024; Sampieri et al., 2025), microvolts (μV) (*n* = 1) (Serafino et al., 2021), and ARV (Gallina et al., 2018b). The pain intensity was reported in eighteen articles using the visual analog scale (*n* = 3) (Abboud et al., 2014; Hao et al., 2020; Arvanitidis et al., 2021), the numerical pain rating scale (*n* = 14) (Madeleine et al., 2006; Falla et al., 2014, 2017; Dideriksen et al., 2016; Gallina et al., 2018b, 2019; Sanderson et al., 2019a,b, 2024; Serafino et al., 2021; Arvanitidis et al., 2022, 2023; Mendez-Rebolledo et al., 2023a; Ducas et al., 2024), and the chronic pain grade questionnaire (*n* = 1) (Sampieri et al., 2025). Only two article did not report pain data (Martinez-Valdes et al., 2019; Li et al., 2024). The average pain intensity varied from 1.8–4.43 (numerical pain rating scale, NPRS) in CLBP subjects and from 4.3–5.5 (NPRS) in upper trapezius experimental pain. Control subjects reported a pain value of 0.

### 3.3 Risk of bias

The evaluation of methodological quality with the adapted Newcastle-Ottawa Scale for cross-sectional studies is shown in Table 4. Three studies presented moderate methodological quality with a total score of 5 stars (Gallina et al., 2019; Mendez-Rebolledo et al., 2023a; Sanderson et al., 2024). The remaining 17 studies presented low methodological quality. All studies included a selected demographic group of participants and only five of them performed a sample size calculation (Arvanitidis et al., 2021, 2022, 2023; Mendez-Rebolledo et al., 2023a; Ducas et al., 2024). Additionally, no study provided information about the response rate of the participants. Ten studies obtained two stars in the item ascertainment of the exposure due to the application of clinical evaluations or validated tools to determine the presence of the clinical pain in the sample (Abboud et al., 2014; Gallina et al., 2019; Martinez-Valdes et al., 2019; Sanderson et al., 2019a,b, 2024; Hao et al., 2020; Serafino et al., 2021; Mendez-Rebolledo et al., 2023a; Sampieri et al., 2025). Regarding the comparability criterion, four studies received a star for including an analysis that addressed potential confounders, such as joint position (Gallina et al., 2018b; Ducas et al., 2024; Sanderson et al., 2024), or for performing multivariate analysis (Gallina et al., 2019). On the other hand, in the outcome items, 15 studies obtained one star in the assessment since they identified the presence of musculoskeletal disorders through a self-reported tool (Abboud et al., 2014; Falla et al., 2014; Gallina et al., 2019; Martinez-Valdes et al., 2019; Sanderson et al., 2019a,b, 2024; Hao et al., 2020; Arvanitidis et al., 2021, 2022, 2023; Serafino et al., 2021; Mendez-Rebolledo et al., 2023a; Li et al., 2024; Sampieri et al., 2025), and four of them were categorized as not applicable because they correspond to studies of experimental pain and not to a diagnosis of clinical pain (Madeleine et al., 2006; Dideriksen et al., 2016; Falla et al., 2017; Ducas et al., 2024). In addition, all studies obtained a star in the statistical analysis item.

**Table 4:** Adapted Newcastle-Ottawa Scale for cross-sectional studies.

**References**	**Selection**				**Comparability**	**Outcome**		**Score**	**MQ**
	**Representativeness of the cases**	**Sample size**	**Non-Response rate**	**Ascertainment of screening/surveillance tools**	**Confounders assessed using subgroup or multivariable analysis**	**Outcome assessment**	**Statistical test**		
Abboud et al. (2014)	SD	-	NR	^**^	NR	^*^	^*^	4	Low
Arvanitidis et al. (2021)	SD	^*^	NR	NR	NR	^*^	^*^	3	Low
Arvanitidis et al. (2022)	SD	^*^	NR	NR	NR	^*^	^*^	3	Low
Falla et al. (2014)	SD	-	NR	NR	NR	^*^	^*^	2	Low
Hao et al. (2020)	SD	-	NR	^**^	NR	^*^	^*^	4	Low
Sanderson et al. (2019a)	SD	-	NR	^**^	NR	^*^	^*^	4	Low
Sanderson et al. (2019b)	SD	-	NR	^**^	NR	^*^	^*^	4	Low
Martinez-Valdes et al. (2019)	SD	-	NR	^**^	NR	^*^	^*^	4	Low
Serafino et al. (2021)	SD	-	NR	^**^	NR	^*^	^*^	4	Low
Arvanitidis et al. (2023)	SD	^*^	NR	NR	NR	^*^	^*^	3	Low
Gallina et al. (2019)	SD	-	NR	^**^	^*^	^*^	^*^	5	Mod
Mendez-Rebolledo et al. (2023a)	SD	^*^	NR	^**^	NR	^*^	^*^	5	Mod
Ducas et al. (2024)	SD	^*^	NR	NR	^*^	NA	^*^	3	Low
Dideriksen et al. (2016)	SD	-	NR	NR	NR	NA	^*^	1	Low
Falla et al. (2017)	SD	-	NR	NR	NR	NA	^*^	1	Low
Madeleine et al. (2006)	SD	-	NR	NR	NR	NA	^*^	1	Low
Gallina et al. (2018b)	SD	-	NR	NR	^*^	NA	^*^	2	Low
Li et al. (2024)	SD	-	NR	^*^	NR	^*^	^*^	3	Low
Sampieri et al. (2025)	SD	-	NR	^**^	NR	^*^	^*^	4	Low
Sanderson et al. (2024)	SD	-	NR	^**^	^*^	^*^	^*^	5	Mod

(-) 0 score; (^*^) 1 score; (^**^) 2 score. SD, selected demographic; NR, not reported; NA, not applicable; MQ, methodological quality; Mod, moderate.

The results of the critical evaluation of studies using sEMG, based on the CEDE checklist (Besomi et al., 2024), are presented in Table 5. Related to the electrode placement section, all of the included studies reported electrode type, muscles evaluated and specified the location of electrodes. Except for three articles (Sanderson et al., 2019a; Hao et al., 2020; Li et al., 2024), all included studies reported the skin preparation procedure, as well as the use and location of the reference electrode. Regarding the electrode characteristics items, all included studies reported the physical configuration of the electrode system, including the type, number, size, and inter-electrode distance, as well as the spatial arrangement of the grids (i.e., 5 × 13, 8 × 8, etc.). Within the items of sEMG signal and its preprocessing section, six studies did not report the signal detection mode (Abboud et al., 2014; Falla et al., 2014, 2017; Dideriksen et al., 2016; Hao et al., 2020; Li et al., 2024). Two of the included studies did not specify the brand and model of the sEMG acquisition system (Hao et al., 2020; Serafino et al., 2021). All included studies specified the gain of amplifier and cut-off frequencies, with the sampling frequency of the sEMG system. All studies reported analog-to-digital resolution and full-scale input range, except for one (Li et al., 2024). Of the total number of studies included, only four did not report the software used for processing the sEMG signal (Martinez-Valdes et al., 2019; Hao et al., 2020; Serafino et al., 2021; Li et al., 2024). Four studies did not report techniques applied for power line interference removal (Hao et al., 2020; Mendez-Rebolledo et al., 2023a; Sanderson et al., 2024; Sampieri et al., 2025). Finally, nine studies used other devices and reported the synchronization with the sEMG system (Gallina et al., 2018b, 2019; Martinez-Valdes et al., 2019; Sanderson et al., 2019a, 2024; Arvanitidis et al., 2021, 2022, 2023; Serafino et al., 2021; Mendez-Rebolledo et al., 2023a; Sampieri et al., 2025). Considering that the design of nine studies did not extract other data at the same time as the sEMG data (Madeleine et al., 2006; Abboud et al., 2014; Falla et al., 2014, 2017; Dideriksen et al., 2016; Sanderson et al., 2019b,a; Hao et al., 2020; Ducas et al., 2024), it was considered that the item of synchronization with other devices did not apply to them.

Table 5Summary of the critical evaluation of studies using electromyography according to the Modified Consensus for Experimental Design in Electromyography (CEDE) checklist.

**Table:** 

**References**	**Electrode placement**					**Characteristics of electrodes**				
	**Muscle**	**EMG type**	**Skin preparation**	**Electrode placement**	**Reference electrode**	**Physical configuration**	**Electrode size**	**Interelectrode distance**	**N electrodes**	**Electrode type**
Abboud et al. (2014)	Yes	Yes	Yes	Yes	Yes	Yes	Yes	Yes	Yes	Yes
Arvanitidis et al. (2021)	Yes	Yes	Yes	Yes	Yes	Yes	Yes	Yes	Yes	Yes
Arvanitidis et al. (2022)	Yes	Yes	Yes	Yes	Yes	Yes	Yes	Yes	Yes	Yes
Falla et al. (2014)	Yes	Yes	Yes	Yes	Yes	Yes	Yes	Yes	Yes	Yes
Hao et al. (2020)	Yes	Yes	No	Yes	No	Yes	Yes	Yes	Yes	Yes
Sanderson et al. (2019a)	Yes	Yes	No	Yes	No	Yes	Yes	Yes	Yes	Yes
Sanderson et al. (2019b)	Yes	Yes	No	Yes	No	Yes	Yes	Yes	Yes	Yes
Martinez-Valdes et al. (2019)	Yes	Yes	Yes	Yes	Yes	Yes	Yes	Yes	Yes	Yes
Serafino et al. (2021)	Yes	Yes	Yes	Yes	Yes	Yes	Yes	Yes	Yes	Yes
Arvanitidis et al. (2023)	Yes	Yes	Yes	Yes	Yes	Yes	Yes	Yes	Yes	Yes
Gallina et al. (2019)	Yes	Yes	Yes	Yes	Yes	Yes	Yes	Yes	Yes	Yes
Mendez-Rebolledo et al. (2023a)	Yes	Yes	Yes	Yes	Yes	Yes	Yes	Yes	Yes	Yes
Ducas et al. (2024)	Yes	Yes	Yes	Yes	Yes	Yes	Yes	Yes	Yes	Yes
Dideriksen et al. (2016)	Yes	Yes	Yes	Yes	Yes	Yes	Yes	Yes	Yes	Yes
Falla et al. (2017)	Yes	Yes	Yes	Yes	Yes	Yes	Yes	Yes	Yes	Yes
Madeleine et al. (2006)	Yes	Yes	Yes	Yes	Yes	Yes	Yes	Yes	Yes	Yes
Gallina et al. (2018b)	Yes	Yes	Yes	Yes	Yes	Yes	Yes	Yes	Yes	Yes
Li et al. (2024)	Yes	Yes	No	Yes	Yes	Yes	Yes	Yes	Yes	Yes
Sampieri et al. (2025)	Yes	Yes	Yes	Yes	No	Yes	Yes	Yes	Yes	Yes
Sanderson et al. (2024)	Yes	Yes	Yes	Yes	Yes	Yes	Yes	Yes	Yes	Yes

**Table:** 

	**EMG signals and pre-processing**							
	**Detection mode**	**Brand/model**	**Gain of amplifier and cut-off frequencies**	**Sampling frequency**	**A/D resolution**	**Software used**	**Power line interference removal**	**Acquisition/synchronization with other devices**
Abboud et al. (2014)	No	Yes	Yes	Yes	Yes	Yes	Yes	NA
Arvanitidis et al. (2021)	Yes	Yes	Yes	Yes	Yes	Yes	Yes	Yes
Arvanitidis et al. (2022)	Yes	Yes	Yes	Yes	Yes	Yes	Yes	Yes
Falla et al. (2014)	No	Yes	Yes	Yes	Yes	Yes	Yes	NA
Hao et al. (2020)	No	No	Yes	Yes	Yes	No	No	NA
Sanderson et al. (2019a)	Yes	Yes	Yes	Yes	Yes	Yes	Yes	Yes
Sanderson et al. (2019b)	Yes	Yes	Yes	Yes	Yes	Yes	Yes	NA
Martinez-Valdes et al. (2019)	Yes	Yes	Yes	Yes	Yes	No	Yes	Yes
Serafino et al. (2021)	Yes	No	Yes	Yes	Yes	No	Yes	Yes
Arvanitidis et al. (2023)	Yes	Yes	Yes	Yes	Yes	Yes	Yes	Yes
Gallina et al. (2019)	Yes	Yes	Yes	Yes	Yes	Yes	Yes	Yes
Mendez-Rebolledo et al. (2023a)	Yes	Yes	Yes	Yes	Yes	Yes	No	Yes
Ducas et al. (2024)	Yes	Yes	Yes	Yes	Yes	Yes	Yes	NA
Dideriksen et al. (2016)	No	Yes	Yes	Yes	Yes	Yes	Yes	NA
Falla et al. (2017)	No	Yes	Yes	Yes	Yes	Yes	Yes	NA
Madeleine et al. (2006)	Yes	Yes	Yes	Yes	Yes	Yes	Yes	NA
Gallina et al. (2018a)	Yes	Yes	Yes	Yes	Yes	Yes	Yes	Yes
Li et al. (2024)	No	Yes	Yes	Yes	No	No	Yes	NA
Sampieri et al. (2025)	Yes	Yes	Yes	Yes	Yes	Yes	No	Yes
Sanderson et al. (2024)	Yes	Yes	Yes	Yes	Yes	Yes	No	Yes

A/D, analog/digital; EMG, electromyography; N, number; Yes, reported; No, not reported; NA, not applicable.

### 3.4 Certainty of the evidence

The results of the analyses, including both clinical and experimental pain, indicate a very low certainty of evidence, downgraded due to inconsistency, indirectness, and publication bias, with a moderate effect size. For clinical pain alone, the evidence was similarly downgraded for the same reasons, with a small effect size, as shown in Supplementary Table S2. The results of the analyses including CLBP show very low certainty of evidence and suggest that higher-quality studies are needed to strengthen it, despite the observation of a small effect size.

### 3.5 Data analysis

#### 3.5.1 Clinical and experimental pain

**Figure 2:**
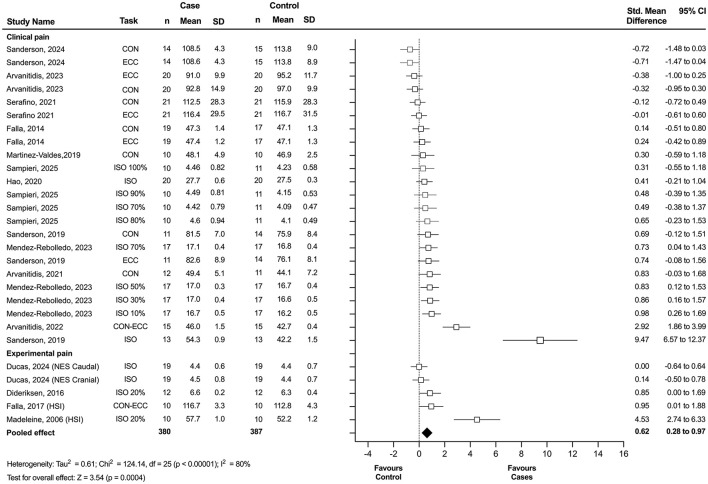
Forest plot showing the displacement of the center of activity for combined experimental and clinical pain. Each study included in the meta-analysis (random-effects model) corresponds to a point estimate with the corresponding 95% confidence interval (CI). The polygon at the bottom of the graph corresponds to the overall effect, and its width represents its 95% CI. Studies with larger squares contributed more to the overall effect size than other studies. Values expressed as percentages represent the relative level of effort with respect to the maximum voluntary contraction. ECC, eccentric task; CON, concentric task; ISO, isometric task; NES, neuromuscular stimulation; HIS, hypertonic saline injection; SD, standard deviation.

Of the 20 studies included in the systematic review, 15 provided sufficient quantitative data on center of activity displacement to be included in the combined meta-analysis of clinical and experimental pain. This analysis revealed a statistically significant displacement of the center of activity in individuals with pain compared to asymptomatic controls, with a moderate effect size (*n* = 28; SMD = 0.62; 95% CI = 0.28 to 0.97; *p* = 0.0004), although these results showed significant heterogeneity (Tau^2^ = 0.61; *p* < 0.00001; I^2^ = 80%) (Figure 2). Due to the lack of homogeneity in sEMG outcomes and the limited number of available articles, it was not possible to conduct secondary analyses for other clinical conditions (e.g., patellofemoral pain syndrome and chronic ankle instability) or experimental pain.

#### 3.5.2 Clinical pain

**Figure 3:**
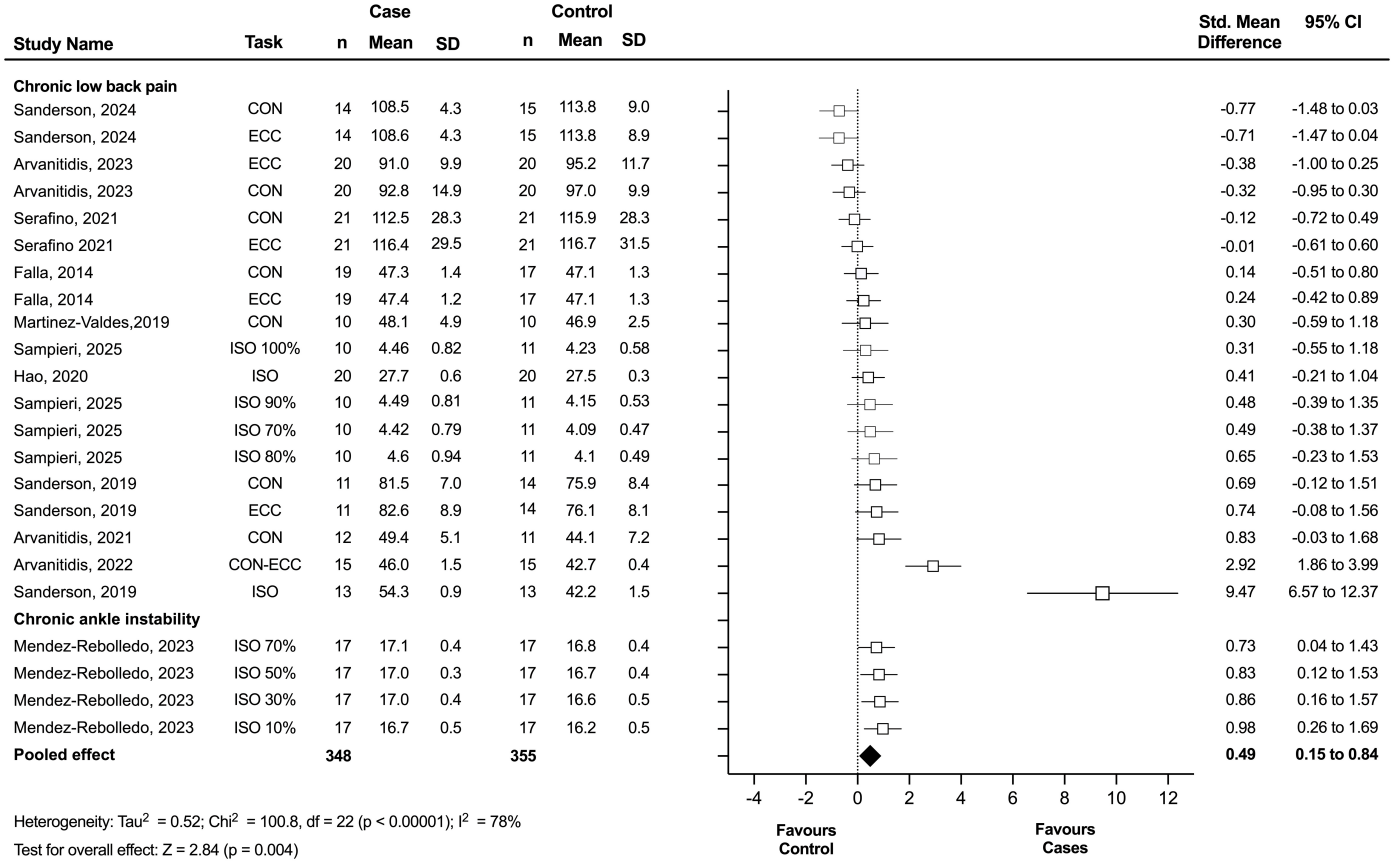
Forest plot showing the displacement of the center of activity for clinical pain. Each study included in the meta-analysis (random-effects model) corresponds to a point estimate with the corresponding 95% confidence interval (CI). The polygon at the bottom of the graph corresponds to the overall effect, and its width represents its 95% CI. Values expressed as percentages represent the relative level of effort with respect to the maximum voluntary contraction. Studies with larger squares contributed more to the overall effect size than other studies. ECC, eccentric task; CON, concentric task; ISO, isometric task; SD, standard deviation.

**Figure 4:**
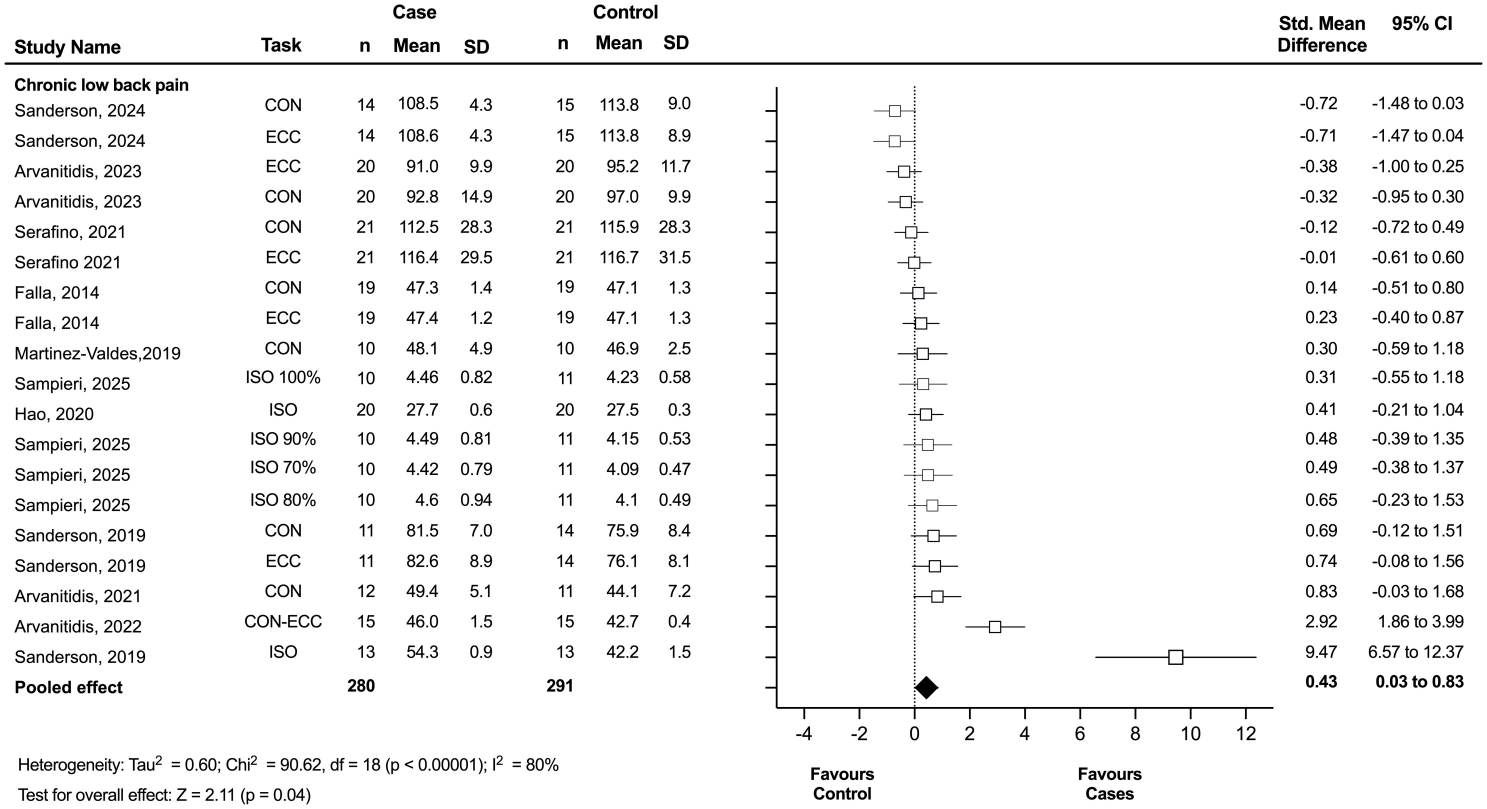
Forest plot showing the displacement of the center of activity for chronic low back pain. Each study included in the meta-analysis (random-effects model) corresponds to a point estimate with the corresponding 95% confidence interval (CI). The polygon at the bottom of the graph corresponds to the overall effect, and its width represents its 95% CI. Values expressed as percentages represent the relative level of effort with respect to the maximum voluntary contraction. Studies with larger squares contributed more to the overall effect size than other studies. ECC, eccentric task; CON, concentric task; ISO, isometric task; SD, standard deviation.

Of the 15 studies included in the meta-analysis, 12 investigated clinical pain populations and were included in the clinical pain analysis. This analysis revealed a statistically significant displacement of the center of activity in individuals with clinical pain compared to asymptomatic controls, with a small effect size (*n* = 23; SMD = 0.49; 95% CI = 0.15 to 0.84; *p* = 0.004), although these results showed significant heterogeneity (Tau^2^ = 0.52; *p* < 0.00001; I^2^ = 78%) (Figure 3). Among these, 10 studies specifically examined individuals with CLBP and were included in the subgroup meta-analysis. This secondary analysis also revealed a statistically significant displacement of the center of activity in individuals with CLBP compared to controls, with a small effect size (*n* = 19; SMD = 0.43; 95% CI = 0.03 to 0.83; *p* = 0.04), although substantial heterogeneity was observed (Tau^2^ = 0.60; *p* < 0.00001; *I*^2^ = 80%) (Figure 4). Notably, studies consistently reported a significant redistribution of erector spinae muscle activity toward the cranial region in individuals with CLBP, as indicated by a marked difference in the location of the center of activity relative to control groups.

#### 3.5.3 Sensitivity analysis

A sensitivity analysis was conducted by excluding the study with the largest effect size (Sanderson et al., 2019b) in each meta-analysis. The results remained consistent with the main analysis for combined clinical and experimental pain (*n* = 27; SMD = 0.46; 95% CI = 0.19 to 0.73; *p* = 0.0008), showing significant heterogeneity (Tau^2^ = 0.35; *p* < 0.01; *I*^2^ = 70%), and for clinical pain alone (*n* = 22; SMD = 0.38; 95% CI = 0.11 to 0.65; *p* = 0.007), also with significant heterogeneity (Tau^2^ = 0.28; *p* < 0.01; *I*^2^ = 66%). However, for CLBP, the sensitivity analysis revealed no statistically significant effects (*n* = 18; SMD = 0.27; 95% CI = −0.04 to 0.57; *p* = 0.09), with significant heterogeneity (Tau^2^ = 0.29; *p* < 0.01; I^2^ = 67%).

## 4 Discussion

This is the first meta-analysis to synthesize evidence on pain-related changes in the spatial distribution of muscle activity, aiming to provide a more robust and generalizable understanding of how chronic musculoskeletal pain and experimental models may alter neuromuscular activation patterns. The main findings indicate a statistically significant difference in the displacement of the center of activity—that is, the spatial distribution of muscle activity—in individuals with clinical pain, including chronic ankle instability, patellofemoral pain syndrome, and CLBP, observed across concentric, eccentric, and isometric muscle contractions. This difference showed a small effect size but was accompanied by a low certainty of evidence due to inconsistency, indirectness, and publication bias. Additionally, the findings were associated with low methodological quality in sample selection criteria and comparability. However, the evaluation of regional muscle activity in the selected studies adhered to current methodological recommendations for sEMG procedures, with most studies meeting the CEDE criteria for electrode placement and reporting of essential characteristics. These results suggest that alterations in the spatial distribution of muscle activity may reflect adaptive neuromuscular responses to chronic pain, potentially influencing motor control strategies. While the center of activity metric does not directly reveal the underlying mechanisms of such shifts, these spatial changes could inform the development of targeted rehabilitation strategies aimed at restoring more balanced activation patterns.

### 4.1 Clinical pain influences regional muscle activity

The alterations in regional muscle activation observed in clinical pain models, as highlighted in the findings of this meta-analysis, may be attributed to several neuromuscular mechanisms. The results suggest a redistribution of muscle activity in individuals with CLBP compared to healthy controls, as indicated by differences in the location of the center of activity. However, this finding was not robust in the sensitivity analysis, highlighting the need for additional studies to validate this effect. A key concept underlying the interpretation of spatial shifts in muscle activation is the phenomenon of “non-uniform motor unit recruitment,” which proposes that pain induces a reorganization of activation patterns within a muscle (Hodges and Tucker, 2011; Hodges and Smeets, 2015; Hodges et al., 2021; Hug et al., 2025). Instead of a uniform reduction in activity across the muscle, some motor units may be inhibited while others are facilitated, resulting in altered spatial distribution of activity (Hao et al., 2020; Arvanitidis et al., 2021). For instance, Hug et al. (2025) demonstrated that during experimental muscle pain, inhibitory inputs are not homogeneously distributed among motor units within the same muscle. By analyzing intrasubject variability, they found that some motor units exhibited significant decreases in discharge rate while others remained unchanged or slightly increased, indicating a non-uniform, task-dependent modulation of motor output. This heterogeneity may reflect an adaptive strategy by the nervous system to redistribute load away from sensitized regions while maintaining overall functional performance (Hug et al., 2025). In addition, previous work has proposed that the effective neural drive to the muscle is primarily governed by the common synaptic input received by the motoneuron pool (Farina and Negro, 2015). More recent findings suggest that biomechanical properties of the muscle, such as twitch duration, can influence how these common inputs are transmitted and expressed, implying that spatial shifts in muscle activity may result from both neural and biomechanical factors that shape how motor commands are distributed across the muscle (Cabral et al., 2024). While this review did not examine within-task temporal variation, the observed between-group differences in the location of the center of activity suggest a stable, pain-related reorganization that manifests during a given motor task. This redistribution could serve an adaptive role, potentially minimizing local tissue stress, redistributing load across muscle regions, or compensating for regional fatigue vulnerability in chronic pain populations (Hodges and Tucker, 2011; Hodges and Smeets, 2015; Hodges et al., 2021). However, whether such changes represent protective strategies or maladaptive compensations remains unclear, and further investigation is needed to explore the physiological mechanisms and functional implications of within-task spatial shifts in chronic pain conditions (Hodges and Tucker, 2011; Abboud et al., 2021).

Experimental studies have shown that nociceptive input can reduce motor unit discharge rates while recruiting additional units to maintain force output (Tucker and Hodges, 2009; Martinez-Valdes et al., 2021). While such evidence is based on pre- vs. post-pain comparisons in controlled settings, how these changes translate to within-task recruitment strategies in individuals with chronic pain remains to be fully elucidated. This recruitment strategy may result in a redistribution of muscle activity either within the same muscle or between synergistic muscles, potentially to unload painful regions or optimize force production under altered conditions (Gallina et al., 2018b; Nuccio et al., 2021). In axial muscles, such as the erector spinae, this redistribution may occur without necessarily altering the global force vector but rather reflect spatial shifts in neural drive across portions of large, multifunctional muscle groups (Abboud et al., 2020). In contrast, in peripheral muscles such as the vasti, changes in recruitment may also influence the direction or orientation of the force vector produced by the muscle (Gallina et al., 2018a,b). Similar adaptations have been reported in chronic musculoskeletal disorders such as CLBP, chronic ankle instability, and patellofemoral pain syndrome. Although not all studies used center of activity metrics, changes in muscle activation patterns, based on signal amplitude or spatial distribution, have been interpreted as evidence of intra- or intermuscular redistribution in response to pain or instability (Gallina et al., 2018b; Arvanitidis et al., 2021; Mendez-Rebolledo et al., 2023a, 2025).

Our meta-analysis revealed a significant redistribution of erector spinae muscle activity toward cranial regions in individuals with CLBP, as indicated by a marked difference in the center of activity location compared to control groups. During isometric and dynamic tasks, the center of activity in people with CLBP tends to shift toward the upper part of the lumbar spine (Sanderson et al., 2019b; Hao et al., 2020; Arvanitidis et al., 2021). This cranial shift may reflect a strategy to adopt a more favorable position for posture control and spinal stabilization, suggesting an effort by the nervous system to shift the load away from potentially affected regions of the lower back. Additionally, previous research has proposed a redistribution of muscle activity from deep to superficial layers of the erector spinae as a strategy to reduce load on injured structures, albeit at the expense of reduced efficiency in spinal stabilization. However, given that HD-sEMG primarily captures superficial muscle activity, such deep-to-superficial shifts are unlikely to be directly reflected in the center of activity measure (Van Dieën et al., 2019; Abboud et al., 2021). This change may be counterproductive in the long term, as excessive activation of superficial and cranial muscle parts can lead to fatigue, deterioration of force steadiness, and alteration of postural control, potentially further aggravating the CLBP condition (Hodges and Tucker, 2011; Arvanitidis et al., 2022). The reorganization of motor recruitment in this case could not only reduce pain but also alter movement dynamics, affecting posture and global motor control (Serafino et al., 2021).

The nature of the motor task also plays a crucial role in these alterations. Tasks that require dynamic movements, different contraction speeds, or prolonged static postures (e.g., isometric contraction) can exacerbate or reveal different activation patterns due to varying demands on the musculoskeletal system (Martinez-Valdes et al., 2021; Arvanitidis et al., 2023; Cruz-Montecinos et al., 2025). For example, in dynamic tasks such as rowing, a caudal shift in the activity of the erector spinae has been reported (Martinez-Valdes et al., 2019). In isometric resistance tasks, fatigue may induce a shift of the center of activity toward more cranial regions, potentially reflecting a strategy by the nervous system to redistribute activation and delay fatigue in areas affected by pain (Hao et al., 2020; Abboud et al., 2021). Additionally, pain induces changes in motor performance, motor unit recruitment, and rate coding behavior that vary across different contraction speeds (Martinez-Valdes et al., 2021). Notably, at higher contraction speeds, the inhibitory effect of pain on lower-threshold motor units is compensated by increased recruitment of higher-threshold motor units, allowing fast submaximal contractions to be maintained. Conversely, at slower speeds, pain reduces motor unit discharge rates and prolongs the neuromechanical delay, which could increase the risk of overload in other muscle regions or adjacent muscles, potentially leading to exacerbation of pain or new injuries.

### 4.2 Experimental pain and regional muscle activity

Although a secondary analysis specifically on experimental pain and its implications for regional muscle activation was not possible, the systematic review of the evidence revealed some key observations. The data suggest that in certain muscle groups, particularly the upper trapezius, there is a caudal shift of the center of activity following pain application by hypertonic saline injection (Madeleine et al., 2006; Falla et al., 2017). However, this redistribution of muscle activity was not observed across all muscle groups. For instance, a caudal shift in the center of activity of the erector spinae muscles was reported in only one study (Dideriksen et al., 2016), and no redistribution was observed in the vastus medialis and lateralis muscles (Gallina et al., 2018b). This suggests that the response may be muscle-specific and influenced by factors such as the nature of the task and the intensity of the painful stimulus (Ducas et al., 2024).

The difference in the shift of the center of activity between experimental and clinical pain models may be attributed to the nature and duration of the painful stimulus. Experimental pain, typically induced acutely by hypertonic saline injections, produces a strong and immediate pain response (Izumi et al., 2014; Christensen et al., 2022; Graven-Nielsen, 2022). In the erector spinae, this acute stimulus may elicit a protective neuromuscular response that shifts muscle activation away from the localized painful region. This has been associated with a caudal shift in activation within the muscle, interpreted as an effort to redistribute loading while maintaining spinal stability. This response could serve as a short-term strategy to minimize discomfort and prevent further irritation during sustained or intense contractions. In contrast, clinical pain, which is often chronic and persistent, likely induces distinct neuromuscular adaptations over time. In conditions such as CLBP, evidence suggests a cranial shift in the activation of the erector spinae muscles. This shift may represent a compensatory mechanism in response to the overload and fatigue that the erector spinae muscles initially endured during the onset of this condition. Holtermann et al. (2011) supports this idea by showing that pain intensity is closely related to the inability to evenly distribute muscle activity in the upper trapezius. The high intensity of pain induced by hypertonic saline in experimental models may contribute to the observed caudal shift in muscle activity in this group.

### 4.3 Clinical implications

This review suggests that individuals with clinical and experimental pain exhibit altered spatial distribution of muscle activity, reflecting potential maladaptive neuromuscular responses to chronic pain. While the clinical implications of these spatial shifts require further investigation, current evidence supports the integration of HD-sEMG as both an assessment and interventional tool in neuromuscular rehabilitation. These findings underscore the need for targeted rehabilitation strategies to promote more effective motor control. Unlike conventional bipolar EMG, HD-sEMG provides a detailed topography of muscle activity, enabling clinicians to detect regional imbalances and monitor neuromuscular adaptations with high spatial precision. This capability is particularly relevant in conditions such as chronic ankle instability, patellofemoral pain, and chronic low back pain, where alterations in motor unit recruitment contribute to recurrent symptoms and functional impairments. Recent studies have demonstrated the utility of HD-sEMG-based biofeedback for retraining the spatial distribution of muscle activity. For instance, Mendez-Rebolledo et al. (2025) used HD-sEMG maps to provide real-time feedback to individuals with CAI, promoting the activation of the under-recruited posterior region of the fibularis longus and restoring a more physiological distribution pattern (Mendez-Rebolledo et al., 2025). Similarly, Arvanitidis et al. (2019) showed that healthy subjects could volitionally modulate the barycenter of trapezius activation using spatial feedback, maintaining a caudal shift in the spatial distribution of muscle activity even under fatigue, highlighting its robustness and applicability during sustained contractions (Arvanitidis et al., 2019). Extending this paradigm, Gazzoni and Cerone (2021) introduced an augmented reality system that projects HD-sEMG-based activity maps directly onto the skin surface via smart-glasses or mobile devices. This immersive visualization allows both patients and clinicians to monitor and adjust muscle activation in real time, improving motor learning through embodied feedback (Gazzoni and Cerone, 2021). In proof-of-concept applications involving lumbar and fibular muscles, this approach revealed asymmetric or maladaptive patterns that were not visible with traditional displays. Collectively, these findings underscore HD-sEMG's potential to guide personalized rehabilitation strategies, enhance patient engagement through intuitive feedback, and objectively quantify progress. Future research should expand beyond observational studies and integrate HD-sEMG with complementary methodologies, such as motor unit decomposition and elastography, to better elucidate the underlying neuromechanical mechanisms. In parallel, testing these approaches across a broader range of clinical conditions (e.g., rotator cuff disorders, cervical pain syndromes, postoperative recovery) and functional contexts (e.g., gait, dual-task balance, or fatigue-inducing tasks) may inform the development of targeted interventions addressing both spatial activation deficits and their functional consequences.

## 5 Limitations and strengths

This study has several limitations that impact the certainty and generalizability of the findings. The combination of small sample sizes and participant variability likely introduced inconsistencies that mask the true nature of neuromuscular adaptations to chronic pain. Additionally, the methodological quality, as assessed by the adapted Newcastle-Ottawa Scale, was generally low, with only three studies achieving moderate quality. The indirectness of evidence, stemming from differences in experimental setups, pain models, and the limited number of muscles investigated, complicates the interpretation of the findings and raises concerns about their applicability across different populations and conditions. Physiologically, motor unit recruitment and muscle activation mechanisms in response to pain may vary depending on the specific chronic condition or muscle group involved, leading to distinct patterns of adaptation that were not fully captured in this meta-analysis. For instance, although a shift in the center of activity was observed in individuals with CLBP, sensitivity analysis indicated that this finding may not be robust. In contrast, for other conditions such as chronic ankle instability or patellofemoral pain syndrome, the limited number of studies prevented firm conclusions regarding the presence or direction of any consistent shift, likely due to their unique biomechanical and functional characteristics. Lastly, although the center of activity offers a convenient, centroid-based summary of the HD-sEMG map, it reduces complex two-dimensional information to two coordinates and is sensitive to several non-physiological factors—including electrode migration relative to the muscle belly, subcutaneous tissue deformation, cross-talk from adjacent or deeper muscles, and movement-related or cardiovascular artifacts, particularly in paraspinal recordings. In addition to these sources of variability, the configuration of signal derivation (monopolar vs. bipolar) also plays a critical role in shaping the spatial representation of muscle activity. Monopolar recordings, commonly used in the included studies, are preferred for estimating spatial distribution and calculating features like the center of activity, as they preserve the integrity of the activation map (Gallina et al., 2022). In contrast, bipolar derivations reduce cross-talk but distort spatial representations by computing differences between adjacent electrodes, leading to lower resolution and misrepresentation of activation shifts. Standardizing monopolar configurations is essential to ensure reliable comparisons across studies.

A further conceptual consideration is the interpretation of spatial distribution of muscle activity. Spatial complexity and displacement of the center of activity represent different but complementary aspects of neuromuscular control. Studies using principal component analysis have shown that people with chronic musculoskeletal pain may present reduced spatial complexity, reflecting a limited diversity of muscle activation patterns (Staudenmann et al., 2014; Gallina et al., 2019). In contrast, displacement of the center of activity indicates a shift in the overall location of muscle activation across the electrode grid. Depending on the context, this shift may reflect either an adaptive redistribution of activity to protect sensitive areas, or a maladaptive response associated with impaired motor control. While spatial complexity captures the variability and richness of muscle recruitment strategies, the center of activity reflects how these patterns are reorganized within the muscle. Therefore, a shift in the center of activity does not contradict reduced spatial complexity but instead highlights a complementary dimension of spatial adaptation of muscle activity.

A key strength of this review lies in its rigorous application of the CEDE checklist, which ensured methodological consistency in sEMG procedures, including electrode placement, configuration, and signal reporting. This promotes the reproducibility and reliability of the included data. However, since the study's conclusions are based on the center of activity, a simplified spatial summary metric, it is important to interpret findings cautiously. This measure does not directly reveal the neuromuscular mechanisms driving spatial shifts, which may also be influenced by factors beyond pain. For instance, variations in muscle fatigability between clinical and control groups could affect motor unit recruitment or firing rates, contributing to observed shifts. In the erector spinae, signals from fiber ends in lower lumbar areas (e.g., L3 or below) may generate non-propagating potentials that distort spatial estimates. To better understand pain-related adaptations, future studies should incorporate complementary methods, such as motor unit decomposition, to clarify the physiological sources of these spatial changes.

## 6 Conclusions

This systematic review and meta-analysis demonstrates that individuals with clinical pain, particularly CLBP, exhibit altered spatial distributions of muscle activity, as quantified by shifts in the center of activity. These findings support the hypothesis that neuromuscular adaptations may occur in the presence of chronic pain. However, it remains unclear whether these adaptations are exclusively pain-induced, as pre-existing differences in muscle fatigability, motor unit recruitment capacity, or muscle fiber characteristics may also contribute to the observed patterns. Therefore, spatial variation in muscle activity should be considered a relevant, but not isolated, component in the evaluation and management of chronic pain conditions. Although methodological variability limited the certainty of evidence, most studies adhered to rigorous sEMG guidelines, enhancing the reliability of the extracted data. Future research should incorporate standardized physiological assessments and complementary techniques, ideally within longitudinal or prospective study designs, to better isolate the influence of pain from pre-existing conditions and other contributing factors.

## Data Availability

The raw data supporting the conclusions of this article will be made available by the authors, without undue reservation.
