# Supplementary material for: Effect of experimental and clinical pain on the spatial distribution of muscle activity: a systematic review and meta-analysis
Source: Front Hum Neurosci. 2025 Jul 10;19:1603807. doi: 10.3389/fnhum.2025.1603807 (PMC12287016; doi:10.3389/fnhum.2025.1603807)
Supplement: Supplementary file 1 [file Table_1.docx]

| **Author** | **Reason** |
| --- | --- |
| Finnerman (2003) | It identifies RMS values ​​but does not generate spatial positioning or topographic displacement in the lumbar region. |
| Yong Hu (2004) | Although a multichannel electrode array was used, the study did not quantify spatial displacement using center of activity metrics. The analysis was qualitative, based on streaming RMS topography, and lacked quantitative spatial coordinates aligned with the inclusion criteria. |
| Gaudreault (2005) | They identified the RMS with multiple bipolar electrodes. However, the analyses were performed individually for each electrode without forming a topographic activation map. |
| Sung (2005) | They used a bipolar electrode on each side, which does not allow for identifying a topographic representation of the activity distribution. |
| Gallina (2018a) | They used high-density electromyography but focused their analysis on the inspection of motor units. They did not consider the topographic representation of the activation center of the muscles being evaluated. |
| Pirouzi (2006) | They used pairs of electrodes in multiple muscles with independent muscle activation analysis, which does not allow identifying the spatial activation behavior. |
| Schabrun (2017) | They used pairs of electrodes at two points in the lumbar region to calculate the RMS independently, which does not allow identifying the spatial activation behavior. |
| Claus (2018) | They used fine-wire electrodes on multiple muscles in the lumbar region and fine-wire bipolar electrodes on the abdominal region to record RMS amplitude. This does not allow for the generation of an activation map. |
| Kubo (2019) | Participants with a history of non-chronic muscle strain were considered for evaluation. |
| Abboud (2021) | Healthy participants were considered pain-free, and no experimental pain was applied. |
| Mendez-Rebolledo (2025) | Considered intervention with feedback for participants, which can condition the spatial distribution of activation. |

**Table S1.** Reasons for excluded studies
