# Supplementary material for: Effect of experimental and clinical pain on the spatial distribution of muscle activity: a systematic review and meta-analysis
Source: Front Hum Neurosci. 2025 Jul 10;19:1603807. doi: 10.3389/fnhum.2025.1603807 (PMC12287016; doi:10.3389/fnhum.2025.1603807)
Supplement: Supplementary file 2 [file Table_2.docx]

**Table S2.** Certainty of the evidence

| **Certainty assessment** | | | | | | | **№ of patients** | | **Effect** | **Certainty** |  |
| --- | --- | --- | --- | --- | --- | --- | --- | --- | --- | --- | --- |
| **№ of studies** | **Study design** | **Risk of bias** | **Inconsistency** | **Indirectness** | **Imprecision** | **Other considerations** | **Case** | **Control** | **Absolute (95% CI)** |  |  |
| **Center of activity in clinical and experimental pain** | | | | | | | | | | | |
| 28 | non-randomized studies | not serious | serious^a^ | serious^b^ | not serious | publication bias strongly suspected^c^ | 380 | 387 | SMD **0.62**  (0.28 to 0.97) | ⨁◯◯◯ Very low^a,b,c^ |  |
| **Center of activity in clinical pain** | | | | | | | | | | | |
| 23 | non-randomized studies | not serious | serious^a^ | serious^b^ | not serious | publication bias strongly suspected^c^ | 348 | 355 | SMD **0.49** (0.15 to 0.84) | ⨁◯◯◯ Very low^a,b,c^ |  |
| **Center of activity in chronic low back pain** | | | | | | | | | | | |
| 19 | non-randomized studies | not serious | serious^a^ | not serious | not serious | publication bias strongly suspected^c^ | 280 | 291 | SMD **0.43** (0.03 to 0.83) | ⨁◯◯◯ Very low^a,c^ |  |

CI: confidence interval; SMD: standardized mean difference. Explanations: a. High heterogeneity; b. Differences between participants; c. High risk of publication
